# Understanding abiotic stress in alfalfa: physiological and molecular perspectives on salinity, drought, and heavy metal toxicity

**DOI:** 10.3389/fpls.2025.1627599

**Published:** 2025-07-31

**Authors:** Muhammad Daud, Haixia Qiao, Shouming Xu, Xue Hui, Muhammad Adil, Yan Lu

**Affiliations:** ^1^ School of Life Sciences, Henan University, Kaifeng, Henan, China; ^2^ College of Environmental Science and Technology, Yangzhou University, Yangzhou, China; ^3^ Ningxia Technical College of Wine and Desertification Prevention, Yinchuan, Ningxia, China

**Keywords:** alfalfa, drought tolerance, salinity stress, heavy metal toxicity, transgenic approaches

## Abstract

Alfalfa (*Medicago sativa* L.), a vital perennial legume forage, has been widely cultivated owing to a variety of favorable characteristics, including comprehensive ecological resilience, superior nutritive value, digestibility, and nitrogen fixation capacity. The productivity traits of alfalfa, particularly its biomass yield and forage quality, are profoundly influenced by a range of abiotic stress conditions. As a common abiotic stress, drought adversely impacts growth and photosynthetic efficiency, accompanied by increased oxidative damage and stomatal closure as a mechanism to minimize water loss; meanwhile, transgenic approaches have been employed to enhance drought resilience by improving antioxidant activity and water-use efficiency. Salinity stress disturbs ionic balance, resulting in sodium (Na^+^) toxicity and the generation of oxidative damage; however, alfalfa cultivars exhibit salinity tolerance through mechanisms such as Na^+^ exclusion, K^+^ retention, activation of antioxidant defenses, hormonal regulation, and the upregulation of stress-responsive genes. In addition, heavy metals pose a significant challenge to alfalfa production, as they impair plant development and disrupt symbiotic nitrogen fixation, but recent studies have highlighted the potential of microbial-assisted phytoremediation in mitigating these detrimental effects. By integrating recent findings, this review highlights the intricate physiological, biochemical, and molecular mechanisms involved in alfalfa’s responses to key abiotic stressors specifically drought, salinity, and heavy metal toxicity. Breakthroughs in genetic modification, notably the development of transgenic lines exhibiting altered expression of stress-responsive genes, offer valuable potential for improving stress resilience. Future research should employ omics approaches, advanced gene-editing and *de novo* gene synthesis to target key regulatory elements responsible for stress adaptation.

## 1 Introduction

**Figure 1:**
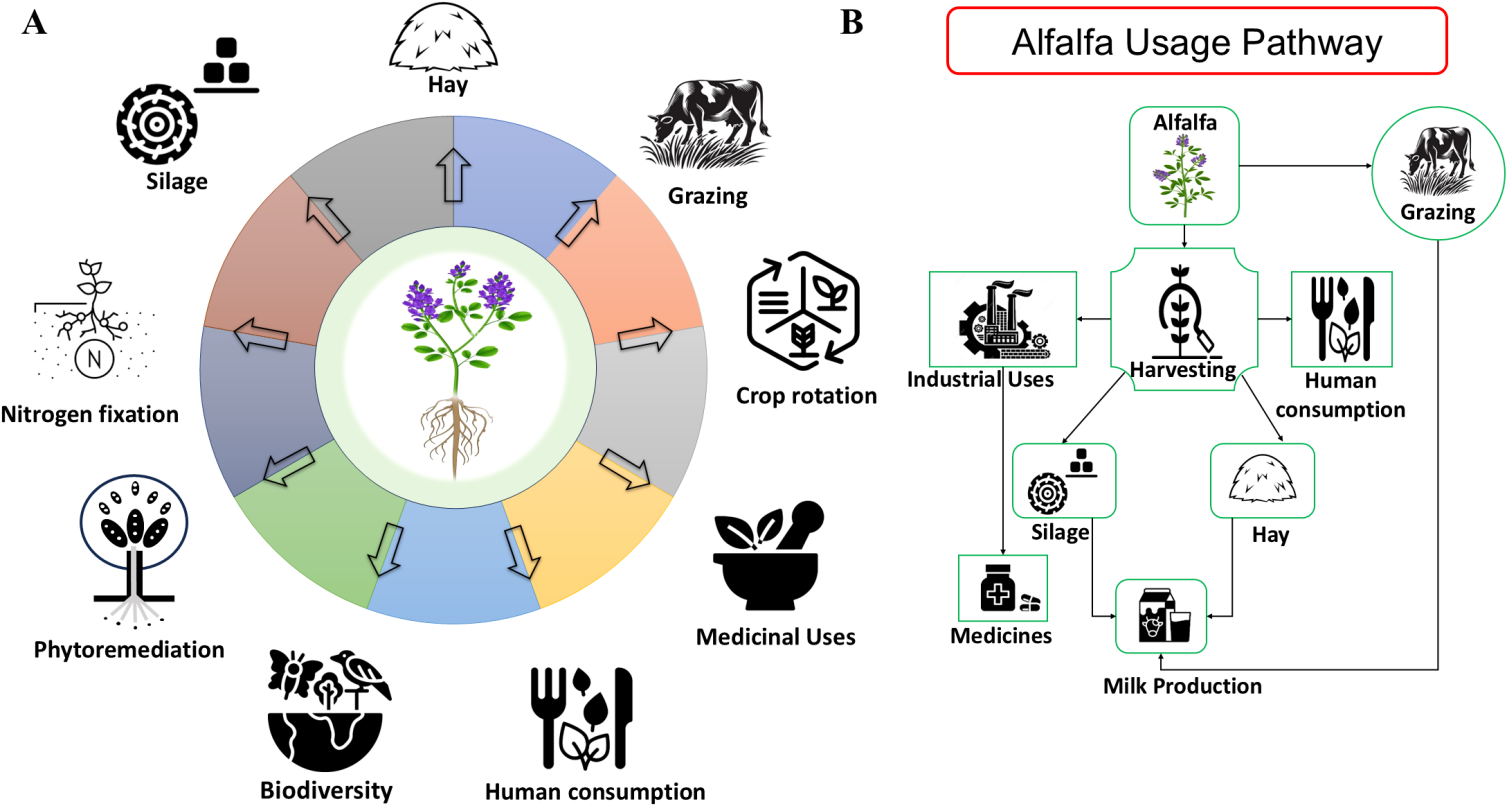
Economic importance of alfalfa. **(A)** Various uses of alfalfa in agriculture, industry, and animal feed. **(B)** Pathways of alfalfa utilization from production to end use.

Alfalfa (*Medicago sativa* L.) is a perennial legume forage that belongs to the subfamily Papilionoideae (Turki and Hegazy, 2021; Steier et al., 2022). Cultivated alfalfa is a cross-pollinated crop and is tetraploid in nature (Hawkins and Yu, 2018). Southwestern Asia is the origin of alfalfa whereas Iran is regarded as its geographic center for this crop (Wang and Şakiroğlu, 2021). In Europe and some other countries, this crop is also called “Lucerne” (Baxevanos et al., 2022). Due to its nutritional value such as high protein content, minerals, carbohydrates, vitamin A, B, C, D and E, it is well known as a staple crop for both humans and animals (Baker et al., 2019; Mattioli et al., 2019; Michalczyk et al., 2019), as depicted in (**Figure 1**). It is one of the oldest plants that was cultivated around 3,300 years ago only for forage purposes with livestock (Michaud et al., 2015). It also acts as a source of essential nutrients, including proteins, vitamins, carbohydrates, and minerals (Hao et al., 2008; Gao et al., 2021). On a dry matter basis, it contains nearly 15 to 22 percent crude proteins along with macro- and trace elements with all the fat- and water-soluble vitamins (Scholtz, 2008).

Alfalfa can also be cultivated in a variety of soil types including poor nutrient soils (Lei et al., 2017). There are many advantages of alfalfa in crop rotation, such as the capability to improve the fertility of soil by nitrogen fixation, as depicted in (**Figure 1**); interestingly, it is found that alfalfa accumulates Nitrogen in large quantities, ranging 300–400 kg/ha/year (Kelner et al., 1997; Angus and Peoples, 2012). Approximately 165 kg/ha of Nitrogen accumulates in the roots and crown, which can be used as a fertilizer for subsequent crops in the same field (Rasse et al., 1999). Generally, this crop is grown for making hay and silage, but because of its high yield and quality of nutrition, it is also used for grazing purposes (Michael and Chandan, 2023), illustrated in (**Figure 1**). In some Chinese and Hindu societies, doctors recommend young leaves of alfalfa for the cure of some disorders such as water retention, arthritis, and digestive tract (Vaibhavi and Devang, 2024). Proper management of alfalfa fields at both local and landscape levels is crucial to maintain the services of the ecosystem, including those dependent on functional biodiversity, and conservation of threatened species (Julier et al., 2017), as shown in (**Figure 1**). Alfalfa can also be used in various recipes including: cooked salad, pudding, souffle’, puree saute’, soup, tea, tortilla, and croquettes (Martínez et al., 2016; Apostol et al., 2017). Some farmers in China regard it as a type of vegetable (Zong et al., 2023) (Djordjević et al., 2024), concluded that alfalfa was used to enhance the mineral, protein, vitamin, and dietary fiber content in wheat flour.

Alfalfa cultivation is profoundly influenced by a range of environmental factors, encompassing both biotic and abiotic stressors, which are responsible for reduction in crop productivity (Wang et al., 2023). Considering the importance of alfalfa, agricultural scientists are paying attention to its cultivation under stressful conditions (Stritzler et al., 2018; Annicchiarico et al., 2022). Drought tolerance in alfalfa is relatively high as compared to that of other forage crops, as alfalfa has a deep root system which ranges from 1.5 to 4m (Huang et al., 2018; Han et al., 2020). Due to the robust rooting system of alfalfa, it regrows successfully (Ali et al., 2021). Alfalfa has been noted to be more drought resistant than other grain legumes (Huang et al., 2018; Han et al., 2020). Drought stress remains a major constraint on alfalfa cultivation, as global temperature rise, evapotranspiration is expected to increase, ultimately worsening drought conditions in arid and semi-arid regions worldwide (Huang et al., 2018). Irmak et al. (2007), concluded that rate of evapotranspiration generally ranges from 0.10 to 0.35 inches per day in alfalfa crops, this level of evapotranspiration supports deep root distribution and high yield (Zhu et al., 2016). Various studies explained drought conditions and their responses through morphologically, physiologically, and biochemically as shown in (**Table 1**).

**Table 1:** Morphological, physiological, and molecular characterization of drought stress tolerance in transgenic and conventional alfalfa.

Targeted gene	Gene functionality	Drought treatment	Putative mechanisms	Experimental conditions	Source
overerpression of γ-tocopherol methyltransferase (*MsTMT*) gene	In the tocopherol biosynthetic pathway, γ-TMT is responsible for catalyzing the production of α-tocopherol	07 days of water restriction using 20% polyethylene glycol (PEG) 6000	**1.** Decreased oxidative damage **2.** Higher water use efficiency and lower stomatal conductance	Controlled conditions	(Ma et al., 2020a)
*MsCYP71*	*MsCYP71* plays key roles in plant growth, development, stress responses	3 weeks of water restriction	Biosynthesis of isoflavonoids and other secondary metabolites that play key roles in defense	Controlled conditions	(Liu et al., 2023a)
*ZxABCG11*	*ZxABCG11* facilitates the transport of cuticular wax components to the aerial surfaces of the plant	withholding of water for 20 days	**1.** Improved biomass yield **2.** Enhanced water retention and photosynthesis capacity	Controlled conditions	(Liu et al., 2023b)
*SPL4-RNAi*	Regulation of Trichome development and regulates the expression of genes responsive to drought	withholding of water for 14 days	Increased root length, water content, chlorophyll content, stomatal conductance, and water potential in leaves	Controlled conditions	(Dan-Dobre, 2022)
Overexpression of *MsWRKY11*	*MsWRKY11* regulates lignin biosynthesis and stomatal density.	3 days of water restriction	**1.** Enhanced water use efficiency. **2.** Decreased Stomatal Density in Leaves.	Controlled conditions	(Wen et al., 2021)
Overexpression of miR156 for *WD40–*1 overexpression	miR156 modulates key plant developmental processes by post-transcriptional silencing of SPL genes	02 weeks of water restriction	**1.** Enhancement of root architecture and photosynthesis efficiency. **2.** Accumulation of primary and secondary metabolites associated with stress response.	Controlled conditions	(Feyissa et al., 2019)
Arabidopsis type I H+ pyrophosphatasegene *AVP1* overexpression	It is necessary for intracellular ions, pH homeostasis, vacuolar cation compartmentation, and overall plant growth.	withholding of water for 35 days	**1.** Taller plants with better growth **2.** Increase in yield biomass **3.** Enhanced the dry root weight as well as root to shoot ratio	Controlled conditions	(Su et al., 2019)
*MsSPL8* down- or up regulation	*SPL8* modulates initiation of axillary bud development, GA signaling, and branching of shoot architecture	2 weeks of water Withholding	**1.** Down-regulation enhanced the crop yield **2.** Suppression of *SPL8* expression prolonged the wilting process. **3.** Down-regulated plants became healthy	Controlled conditions	(Gou et al., 2018)
*HaHB11* expression	Increased yield biomass with better growth as well as flooding tolerance by a quiescent method	Water shortage of 10 days	**1.** Closing of stomata faster **2.** Efficient use of water with Lower water loss	Controlled conditions	(Cabello et al., 2017)
Overexpression of *mi156* (*miR156OE*) and suppression of *SPL13*	Control multiple traits including plant biomass yield, development of seed, fruit, root development and tolerance to abiotic stress	Withholding of water for 15 days	**1.** Reduction of water loss with high survival, more and denser adventitious roots. **2.** Enhanced levels of antioxidants, abscisic acid, photosynthetic assimilation and chlorophyll contents	Controlled conditions	(Arshad et al., 2017)

Several researchers have developed a range of transgenic alfalfa cultivars with enhanced drought tolerance, achieved through the introduction of one or more genes from a single species into another using genetic engineering techniques, including Agrobacterium-mediated transformation or direct gene transfer methods (Gao et al., 2024). Ma et al. (2020a) found that alfalfa resistance to drought stress was improved by the overexpression of the γ-tocopherol methyltransferase gene by alleviated oxidative damage, maintained high water-use efficiency or by lowered the stomatal conductance. Silencing of *SPL13* and overexpression of *miR156* allowed the alfalfa to become tolerant against drought stress (Arshad et al., 2017), while (Feyissa et al., 2019) successfully used moderate expression of *miR156*, which improved the ability of alfalfa to withstand drought through *WD40–*1 overexpression.

Excessive salt accumulation in the soil is also a major limiting factor for crop productivity (Yadav et al., 2019; Hao et al., 2021). As saline soil contains an excess of soluble salts including calcium, sodium, magnesium, chloride, potassium, and sulfate in their root zones, as a result plants fail to absorb nutrients and water from the soil and causes plant injury (MaChado and Serralheiro, 2017; Behdad et al., 2021). Interestingly, alfalfa is also considered a moderately salt-tolerant legume crop (Bertrand et al., 2015). Usingconventional breeding techniques, different cultivars of alfalfa have been developed with salt tolerance (Sandhu et al., 2017); however, attaining salt tolerance in this crop through genetic engineering is very difficult, moreover the response is genetically and physiologically complex against the salt stress because multiple genes are used in controlling salt tolerance including both physiological and biological mechanisms (Smethurst et al., 2008; Hrbáčková et al., 2020). Comprehending salt resistance pathways and detecting genetic traits suitable for evaluating improved salinity tolerance, play a vital role in alfalfa breeding programs (Kaundal et al., 2021). It is essential to identify the genes responsible for salt tolerance in alfalfa crops for the development of molecular markers, precise screening and advancements in plant breeding and genetics (Bhattarai et al., 2021). Recent technologies used in alfalfa salt stress research include RNA-Seq analysis, salt-resistant breeding, and cutting-edge Synchrotron beamlines (Peng et al., 2025).

Heavy metals stress is also a significant concern to discuss after drought and salt stress (Anwar et al., 2021). Human industrialization and agricultural activities lead to environmental contamination and ultimately affecting plant quality and biomass, so it is very important to study the contaminants that are harmful to plants growth (Raza Altaf et al., 2021; Adil et al., 2024a; Ahamad et al., 2024; Razzaq et al., 2024b, 2024a). All harmful substances released into the biosphere have an impact on different types of living organisms, including plants (Migda et al., 2024). These toxic substances (heavy metals) create problems not only for plant health but also for soil integrity. The use of contaminated crops for food and feed, poses threats to human health globally (Bandyopadhyay et al., 2015; Agnello et al., 2016; Gan et al., 2020). As a leguminous plant, alfalfa forms a symbiotic relationship with Gram-negative soil bacteria of the genus *Rhizobium*, both experience detrimental effects due to the presence of heavy metals (HMs) because HMs reduce the symbiotic capacity and ultimately the capacity of alfalfa to fix nitrogen (Li et al., 2014). Recent work done on protection of plants and environment, focused on mitigating the detrimental impact of pollution on plants and soil; however, it has led to the emergence of a relatively recent approach known as stress mitigation, which involves applying external phytochemicals and microbial agents to enhance plant homeostasis or make the plant tolerant against different stresses caused by environment (Jócsák et al., 2022). Overall, this review provides an in-depth understanding of how alfalfa responds at physiological, biochemical, and molecular levels to major abiotic stress factors, specifically drought, salinity, and heavy metal exposure, aiming to support the development of stress-resilient cultivars and guide improved cultivation strategies under such stressful conditions.

## 2 Drought stress

### 2.1 Effect of drought stress on alfalfa growth

A decline in water supply restricts the plant’s nutrient uptake, leading to slower growth and reductions in various growth parameters such as plant height, biomass accumulation (fresh and dry weight), branching intensity, leaf production per plant, leaf area, cell wall thickness in leaves, stomatal density, cutinization of leaf surface, formation of defective vascular tissue**—**as well as premature leaf senescence (Singh et al., 2021; Zia et al., 2021; Adil et al., 2022b, 2022c, 2024b, 2024c). Partial closing of stomata has been noted an early response to water scarcity to reduce water loss through transpiration, however, it also limits photosynthesis and carbon assimilation (Devi and Reddy, 2020). The reduction in transpiration rate due to stomatal closure can improve the water-use efficiency but it negatively affects the transport and uptake of nutrients (Ranawana et al., 2021). Many studies on drought stress have demonstrated that stomatal closure can significantly lessen the negative effects of drought stress in alfalfa crops (Arshad et al., 2017; Luo et al., 2022). In response to water scarcity conditions, non-stomatal mechanisms may include decreased carboxylation enzyme activity, a decline in ATP (adenosine triphosphate) production, and structural damage to the photosynthetic system (Zhang et al., 2019).

The results indicated that drought stress negatively affected alfalfa plants by reducing morphological growth (by 12 to 54%), gas exchange efficiency (by 37 to 88%), and chlorophyll content (Chl *a* and Chl *b* declined by 29% and 40%, respectively), along with reducing mineral content; furthermore, it increased lipid peroxidation by 69% and increased the accumulation of reactive oxygen species (ROS) (Roy et al., 2021). The findings also revealed that plants experiencing drought stress exhibit decreased plasma membrane permeability and stomatal conductance while limiting malondialdehyde accumulation, and increasing proline levels and related hormones, which ultimately strengthens their drought resistance (Yasmin et al., 2021, 2022). The experiment demonstrated that increasing drought stress in alfalfa plants resulted in a significant rise in H_2_O_2_, O_2_^-^, and malondialdehyde levels by 323%, 247%, and 235% respectively, while the enzymatic activities of superoxide dismutase (SOD), catalase (CAT), and Ascorbate Peroxidase (APX) also increased by 18.01%, 15.56%, and 587% under 15% PEG (polyethylene glycol-6000) treatment (Chen et al., 2021). Further research concluded that drought stress in alfalfa plants led to variations in hormone levels such as (Gibberellin (GA_3_), Zeatin (ZA), Abscisic acid (ABA), indole-3-acetic acid (IAA) levels, where GA_3_, ZA, and the GA_3_/ABA ratio reached their highest levels under moderate stress, whereas IAA and IAA/ABA dropped significantly under severe stress, accompanied by an increase in ABA (Wang et al., 2024).

### 2.2 The molecular mechanisms of drought tolerance in alfalfa

Efforts to increase alfalfa stress tolerance under varying growing conditions has recently focused on physiological responses, metabolic activities, morphological adaptions, and genetic modification (Song et al., 2019). Alfalfa demonstrates superior drought resistance over many forages as a result of its deep-penetrating roots (1.5–4.0 m) (Huang et al., 2018; Han et al., 2020). Although chlorophyll content and the rate photosynthesis decline under water scarcity conditions although maintaining chlorophyll under such conditions is associated with better drought resilience (Rokebul Anower et al., 2017). It has been proposed that enhanced stomatal conductance and restricted water loss during drought contribute to the maintenance of higher chlorophyll content, thereby reinforcing drought tolerance in plants (Zheng et al., 2017; Iqbal and Yaning, 2024). Studies suggest that the enhanced drought resilience of alfalfa is closely associated with the accumulation of both organic and inorganic osmolytes (Shanker et al., 2014). Among these, Proline is one of the most well-studied osmolytes in plants like alfalfa, owing to its essential function in preserving leaf relative water content under low water potential, thereby boosting drought resilience (Ni et al., 2012). Legume plants experience a decline in both nodule formation and biological nitrogen fixation under drought conditions (Dollete et al., 2024). Therefore, sustained nitrogen fixation under water-deficit conditions has been linked to increased drought resilience in plants (Xu et al., 2012). Plant breeding approaches, including both traditional methods and genetic engineering, have exhibited considerable potential in strengthening plant tolerance against abiotic challenges (Villalobos-López et al., 2022).

**Figure 2:**
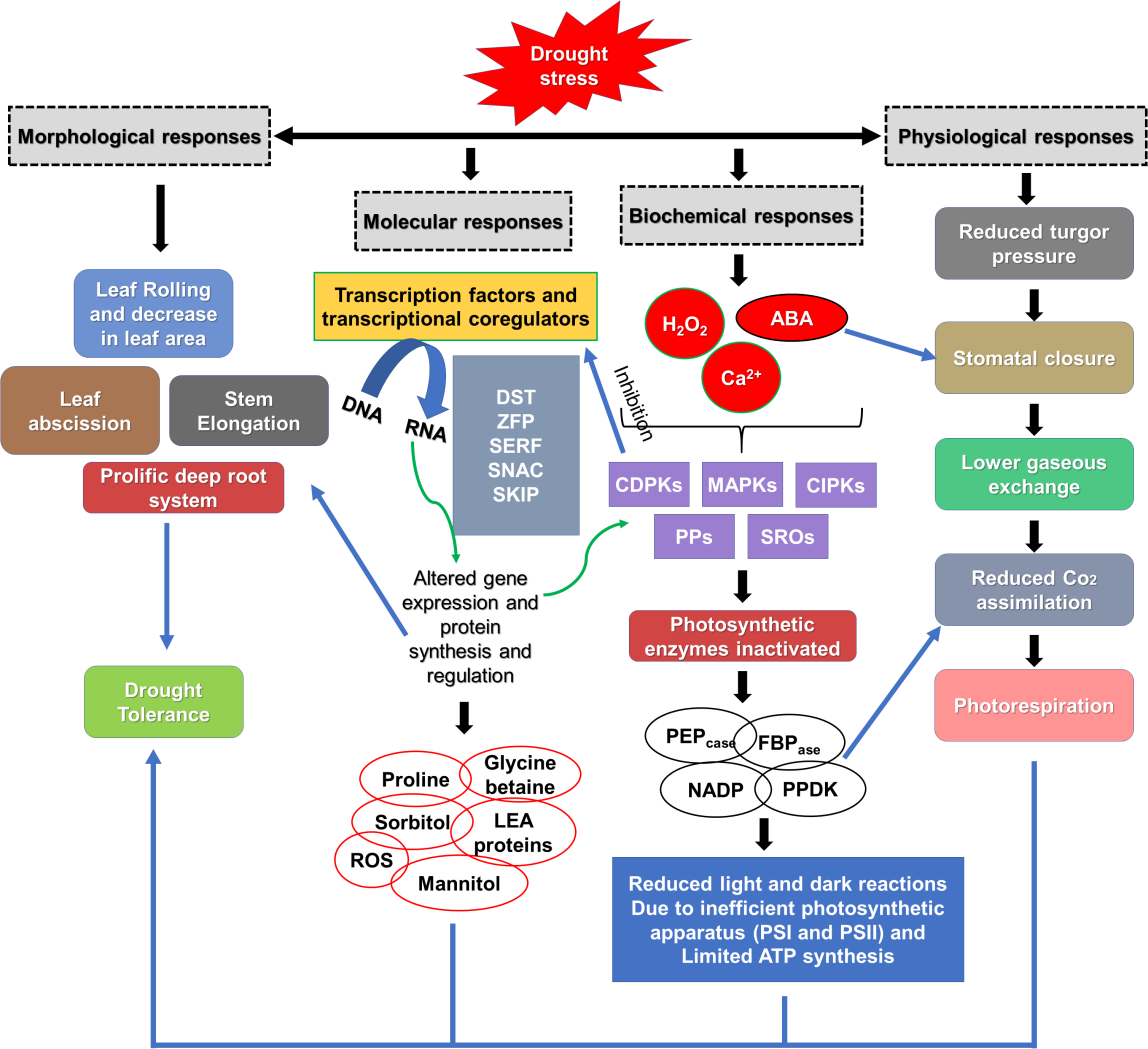
Plant mechanisms to overcome drought conditions. SERF, serum response factor; DST, drought and salt tolerant; SKIP, Ski-interacting protein; ZFP, zinc finger transcription factor; SNAC, stress responsive NAC transcription factor; LEA, late embryogenesis abundant proteins; ROS, reactive oxygen species; SROs, similar to RCD-ONE; CDPKs, calcium dependent protein kinases; CIPKs, CBL interacting protein kinases; PPs, protein phosphatases; MAPKs, mitogen activated protein kinases; ABA, abscisic acid; NADP-ME, NADP malic enzyme; PEPcase, phosphoenol pyruvate carboxylase; PPDKs, pyruvate phosphate dikinases; FBPase, fructose 1,6-bisphosphatase. Preproduced from Zargar et al. (2017) Copyright 2017 Elsevier.

A Numerous genes have the potential to encode transcriptional regulators, including zinc finger proteins (Tang et al., 2013) and NAC transcription factors (Min et al., 2020) associated with stress responses (**Figure 2**). Certain compounds such as proline, glycinebetaine, LEA proteins, abscisic acid and other anti-oxidants are synthesized and over expressed to maintain osmotic balance and protect the structural integrity of the cell under drought stress (Banerjee and Roychoudhury, 2016). Moreover, certain families like MAPK, CDPK, and antioxidants can be a direct or indirect target to enhance drought tolerance (Puri, 2019), (**Figure 2**). Overexpression of PEPcase, pyruvate orthophosphate dikinase (PPDK), NADP-malic enzyme (NADP-ME), and NADP-malate dehydrogenase (NADP-MDH) from *Medicago sativa* L. enhanced alfalfa tolerance by increasing photosynthetic efficiency and promoting nodule formation (Luo et al., 2024). To enhance drought stress tolerance more effectively, numerous transgenic alfalfa plants with enhanced resilience have been developed by various scientists, as shown in (**Table 1**). Overexpression of the γ-tocopherol methyltransferase gene showed greater alfalfa drought resistance by mitigating oxidative stress, inducing the accumulation of osmoregulatory compounds, modulating stomatal conductance, and optimizing water use efficiency in comparison to untreated plants (Ma et al., 2020a). Studies found that overexpressing *miR156* and suppressing *SPL13* can effectively enhance drought stress tolerance in alfalfa (Arshad et al., 2017). Research also concluded that a moderate expression of *miR156* contributed to alfalfa drought resistance through the upregulating of *WD40–1* (Feyissa et al., 2019). The study demonstrated that overexpression of *MsNTF2L* (*M*. *sativa NUCLEAR TRANSPORT FACTOR 2-LIKE*) is a key regulator of drought tolerance in alfalfa; furthermore, scientists determined that it enhanced drought resistance by promoting ROS scavenging, decreasing stomatal density, improving stomatal closure in response to ABA, and increasing the accumulation of epicuticular wax crystals (Luo et al., 2022). Overexpressing *MsTHI1* (*Medicago sativa Thiamine Thiazole Synthase 1*) improved drought resistance through enhanced levels of vitamin B1, chlorophyll *a* (Chl a), chlorophyll *b* (Chl b), enhanced antioxidant activity, photosynthetic efficiency, signal transduction, and the activation of stress-related genes (Yin et al., 2022). Additionally, it is found that the bacterial strain *DGL1* enhanced alfalfa’s drought resistance through the production of extracellular polysaccharides, deaminase, and solubilizing phosphorus (Yang et al., 2024).

## 3 Toxicological effects of heavy metals on alfalfa’s growth patterns

### 3.1 Influence of essential heavy metals on alfalfa growth

Zinc (Zn): High concentration of Zn results in leaf chlorosis, inhibition of growth and reduction in photosynthetic rate due to Zn toxicity (Reddy and Kumari, 2022) (Bandyopadhyay et al., 2015), concluded that an excess level of Zn (750 mg/kg soil) accumulatesin the root zones, approximately 300–400 mg kg^-1^ DW. Research conducted by (Ibekwe et al., 1996), stated that treatment with 4–7.3 mM Zn after 10 days of exposure resulted in chlorotic symptoms with poor root development and necrotic spots. Yahaghi et al. (2019), showed that Zn treatment with 1.5–24 mM Zn affected the rate of germination.

Manganese (Mn): (Li et al., 2019), investigated the symptoms of Mn toxicity as interveinal chlorosis found in mature leaves, roots browning, nutrient uptake disruption, necrotic spots found in mature leaves. (Sale et al., 1992) summarized the effect of Mn toxicity on alfalfa with approximately (60 mg L^-1^) resulted 20% less dry weight as compared to plants in control (Gherardi and Rengel, 2003), summarized the Mn symptoms with 500 mg g^-1^ and noticed a reduction in roots as well as shoots of alfalfa plants.

Nickel (Ni): Nickel is considered an essential heavy metal because of its presence in glyoxalase enzymes, the porphyrin compound F430, peptide deformylases, and because it acts as a central metal atom of some hydrogenases and superoxide dismutases (Dixon et al., 1975). Alfalfa is capable of absorbing nickel, after sixty days of exposure with Ni (0, 50, 150, 250, and 500 mg kg^-1^) resulted an increase in MDA levels and the activities of glutathione-S-transferase (GST) and peroxidase (POX); whereas GST, phytochelatin synthase (PCs) and *Prx1C* were also upregulated in roots and shoots of alfalfa (Helaoui et al., 2020) (**Table 2**).

Copper (Cu): Diazotrophic bacteria are restricted to grow and reproduce due to the presence of copper, which is responsible for the fixation of nitrogen in alfalfa plants (Sharaff and Archana, 2016). when copper is present in high amounts, it accumulates in the stem apoplasts of plant, then it influences the properties of cell wall and ultimately affects the alfalfa quality; furthermore, it leads to a reduction in ion concentration in alfalfa stems and reduces the concentration of ferritins—ubiquitous proteins that regulate the amounts of Fe in the redox state of cells (Strozycki et al., 2010).

### 3.2 Adverse impacts of non-essential heavy metals on alfalfa

Lead (Pb): Alfalfa plants exposed to pb, showed symptoms of chlorosis, reduced growth and reduction in photosynthetic rate (Yan et al., 2010) (Lopez et al., 2007), performed an experiment in alfalfa plants which were exposed with 40 mg/L of lead and concluded that activity of CAT decreases, but total amylase activity (TAA) increases in alfalfa leaves. (Hattab et al., 2016) conducted research to measure the amount of stress in alfalfa by applying Pb with 0, 10, and 100 mM for 2 and 7 days; furthermore, he observed a reduction in levels of homoglutathione (hGSH) as well as root glutathione (GSH). Research concluded that root growth and development were hindered by lead toxicity, disrupted the early stages of the legume-Rhizobium symbiotic relationship and affected the biochemical signaling involved (Besharati and Memar, 2017). The accumulation of heat shock proteins such as HSP70 and HSP17.7 was found to be higher in plant shoots, reflecting that lead toxicity triggered protective cellular responses against lead stress (Hattab et al., 2016).

Cadmium (Cd): In some crops the toxicity of Cd affects the uptake of water and nutrients (Andresen and Küpper, 2013). In alfalfa, Ca, Fe, Mg, and K contents were decreased by Cd concentrations at 3 and 5 mg/kg soil; furthermore, reduction was also reported in dry matter, root and shoot length (Dražić et al., 2006). It not only affects the process of germination, but also affects the growth of seedling after germination (Yahaghi et al., 2019). When Cd is exposed to alfalfa plant, it also affected the physiological, morphological functions as well as metabolism (Dražić et al., 2006; Haider et al., 2021). Cd also exhibits negative effects on photosynthesis, oxidative stress, root metabolism inhibition, and genotoxicity (Andresen and Küpper, 2013).

Chromium (Cr): Hexavalent chromium [Cr (VI)] exhibits solubility within the pH range of natural water, and can be found in irrigation water, it is considered a toxic metal for aquatic and terrestrial (Salmani Abyaneh and Fazaelipoor, 2016). Study concluded that hexavalent chromium [Cr (VI)] exposed at 5 and 10 mg L^-1^ K_2_Cr_2_O_7_, reduced the size of leaf, number of photosynthetic pigments, reduction of biomass, but increasing SOD, NO, H_2_O_2_, and CAT activities, which were partially maintained through the transcriptional regulation of Cu/ZnSOD, FeSOD, MnSOD and CAT genes (Christou et al., 2020) (**Table 2**).

Mercury (Hg): Mercury toxicity hinders the growth and development of alfalfa while also disrupting iron and sulfur balance and promoting oxidative stress (El-Shehawi et al., 2022). By applying Hg with a quantity of 4, 5, 10, 20, and 40 M along with an exposure of O_2_^-^ and H_2_O_2_ generation in leaves of alfalfa plant and recorded increase in lipoxygenase (LOX), POD, NADH-oxidase, APX, and CAT activities (Zhou et al., 2008, 2009). Findings showed that mercury exposure in alfalfa plants resulted in increased lipid peroxidation, reduced chlorophyll levels, and impaired glutathione reductase (GR) activity in roots, as well as the production of a new root peroxidase isoform, reflecting redox imbalance (Sobrino-Plata et al., 2009).

**Table 2:** Heavy metal exposure in alfalfa: affected parts, concentration levels, and duration of stress.

Plant Part	Heavy Metal Exposure	Altered Physiological Processes	Metal uptake by Plant	Treatment Duration	Source
Whole plant	Mn: 60 mg L^-1^	20% reduction in dry weight	N. A	35 days	(Li et al., 2019)
Seeds	Zn applied with 1.5–24 mM	Reduction in rate of germination	Zn: root: 490 mg kg^-1^ Zn: shoot: 180 mg kg^-1^	24 h	(Yahaghi et al., 2019)
Seeds	Pb applied with 1.5–24 mM	Reduction in rate of germination	Pb: root: 1330 mg kg^-1^ Pb: shoot: 300 mg kg^-1^	24 h	(Yahaghi et al., 2019)
Roots	Cd: 1 mM	Decline in soluble proteins, enzymatic activity, enhanced electrolyte leakage, up-regulated three Fe- related genes: *MsIRT1*, *MsNramp1*, *MsFRO1*	root: 10 mg kg^−1^ DW	7 days	(Kabir et al., 2016)
Roots	Cd: 0–40 µM	Tolerant genotypes: enhanced cadmium accumulation, dry biomass, germination efficiency, reduced lipid peroxidation and improved plasma membrane stability	600–1450 mg kg^-1^ DW in non-resistant cultivars whereas 600–1700 mg kg^-1^ DW in stress resistant cultivars	48h, 72h, 96h	(García de la Torre et al., 2021)
Roots	Ni applied with 0, 50, 150, 250, 500 mg kg^-1^	Increasing POX, MDA level and GST activities	0.61; 1.96; 9.97; 11.68; 23.65 mg kg^-1^ DW respectively	60 days	(Helaoui et al., 2020)
Roots	(Pb) applied with 0, 10, 100	Reduced the levels of hGSH and GSH, enhancement in lipid peroxidation, APX, HSPs and GR levels	766.66 mg Kg^-1^ DW	2 days	(Hattab et al., 2016)
Shoots	Ni: 50; 150; 250; 500 mg kg^−1^	Enhanced POX, GST, MDA levels, and up-regulation of *Prx1C*, *GST* and *PCs* genes	DW: 1.58; 8.92; 22.64; 32.84; 75.2 mg kg^−1^	60 days	(Helaoui et al., 2020)
Shoots	Cd: 1 mM	Decreased soluble proteins, enzymatic activity, enhanced electrolyte leakage, up-regulated three Fe- related genes: *MsIRT1*, *MsNramp1*, *MsFRO1*	DW: 1.4 mg kg^−1^	7 days	(Kabir et al., 2016)
Shoots	Cd: 0–40 µM	Tolerant genotypes: enhanced cadmium accumulation, dry biomass, germination efficiency, reduced lipid peroxidation and improved plasma membrane stability	DW: 25–31 mg kg^−1^ in resistant and non-resistant cultivars	48; 72; 96 h	(García de la Torre et al., 2021)
Root Cotyledon Leaves	Pb	Stunted growth, chlorosis, and low photosynthetic rate	DW: Cotyledon: 300 mg L^−1,^ Root: 25,500 mg L^−1^, Leaves: 29 mg L^−1^	50 days	(Yan et al., 2010)
Leaves	Cr: 0.05; 0.5; 1; 5; 10 mg L^−1^	Reduction in biomass, leaf size, photosynthesis, increase of lipid peroxidation, and ROS	DW: 2.5; 2.8; 5; 8; 16 mg kg^−1^	59 days	(Christou et al., 2020)

### 3.3 Heavy metals tolerance

**Figure 3:**
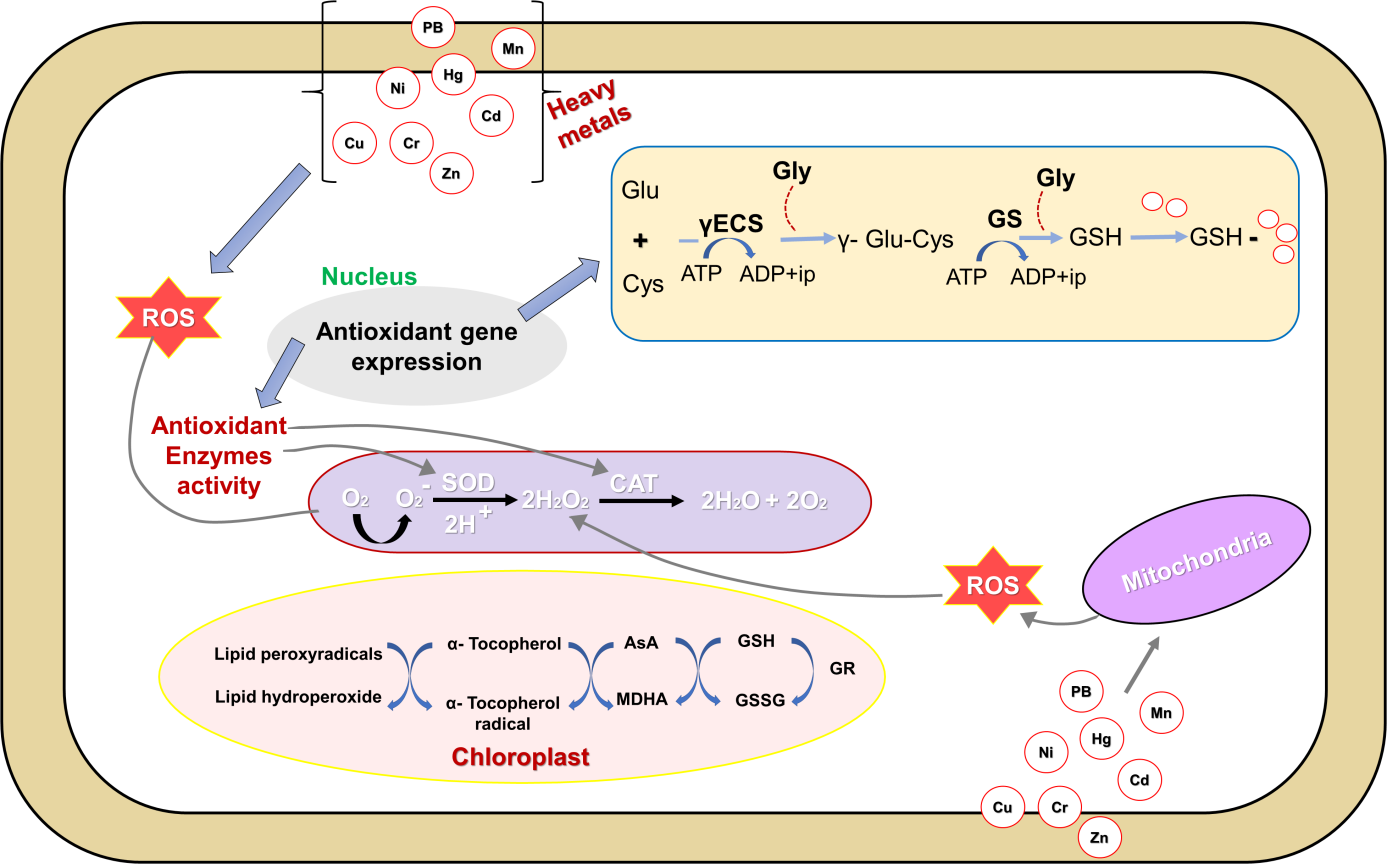
Mechanisms of oxidative stress, tolerance, and detoxification in plant cells under the exposure of heavy metals. ROS, reactive oxygen species; O^−^_2_, superoxide radicals; O_2_, oxygen molecule; H_2_O_2_, hydrogen peroxide; CAT, catalase; SOD, superoxide dismutase; AsA, ascorbic acid; GSH, glutathione (reduced); MDHA, monodehydroascorbate; GSSG, oxidized glutathione; GR, glutathione reductase; Glu, glutamine; Cys, cysteine; GS, glutathione synthetase; Gly, glycine; Pb; Mn; Hg; Ni; Cd; Cu; Cr, Zn: Heavy metals. Reproduced from Singh et al. (2016) Copyright 2016 Frontiers Media SA.

The cellular redox balance under heavy metal stress is maintained through the prompt quenching of reactive oxygen species by a coordinated action of enzymatic (SOD: superoxide dismutase, CAT: catalase, APX: ascorbate peroxidase, GR: glutathione reductase, MDHAR: monodehydroascorbate reductase, DHAR: dehydroascorbate reductase, GPX: glutathione peroxidase, and glutathione-S transferase) as well as non-enzymatic (ascorbate, glutathione, proline, and α-tocopherol) antioxidant defense systems (Singh et al., 2016), (**Figure 3**). Glutathione (GSH), as a low-molecular-weight, water-soluble tripeptide (γ-Glu-Cys-Gly), functions as a critical component of the cellular defense system is crucial in mitigating the toxic effects of heavy metal exposure (Flores-Cáceres et al., 2015). Glutathione reductase (GR) efficiently catalyzes the conversion of oxidized glutathione (GSSG) back to its reduced form (GSH), and possesses a conserved disulfide linkage that is susceptible to disruption under metal-induced stress (Hajiboland et al., 2015), and contributes significantly to cellular defense by facilitating the reduction of GSSG, thereby sustaining a high GSH to GSSG ratio essential for redox homeostasis (**Figure 3**). Surprisingly, the role of *arbuscular mycorrhizal* (AM) fungi to tolerate and accumulate heavy metals including nickel, lead, cadmium, mercury, chromium, and arsenic has been widely recognized in scientific studies (Helaoui et al., 2020; Boorboori and Zhang, 2022). The study revealed that lead (Pb) stress hindered plant growth and disrupted photosynthesis, but the presence of AM fungi (*Glomus intraradices*) helped mitigate these harmful effects (Kahromi and Najafi, 2020). Results indicated that inoculating seedlings with the bacterial species (*Sinorhizobium meliloti*) alleviated growth suppression caused by copper stress and enhanced nitrogen uptake in seedlings, leading to an overall increase in plant nitrogen concentration (Chen et al., 2018; Duan et al., 2022). The application of silicon to plants under Cd stress significantly improved their morpho-physiological characteristics, increased total protein levels, and maintained membrane integrity, highlighting silicon’s crucial role in alleviating Cd-induced stress (Kabir et al., 2016). The NT27 isolate as a strain of *Pseudomonas* sp. significantly boosted *Medicago sativa* growth, increasing shoot dry weight (97.6%) and root dry weight (95.4%) under chromium stress; furthermore, it also enhanced chlorophyll content, reduced stress markers, and promoted Phytostabilization in plants (Tirry et al., 2021). Research revealed that plants inoculated with a *Rhizobium tibeticum* strain at a 0.005 mM Ni concentration led to a notable increase in nodule formation, root length, shoot length, and shoot dry mass compared to non-inoculated alfalfa plants under Nickel stress (Pešić et al., 2025).

## 4 Salt stress

### 4.1 Impact of salt stress on the growth and development of alfalfa

Excessive accumulation of soluble salts, including chloride, sulfate, and carbonate compounds of key cations like sodium, calcium, magnesium, and potassium, significantly disrupts the efficiency of water and nutrient acquisition by plants (Bhattarai et al., 2020; Liu and Wang, 2021). In severe conditions of salt stress, the nature of soil solution becomes hyper-osmotic, which is responsible for leading to water loss, as a result, plants experience wilting and premature senescence (An et al., 2016; Long et al., 2019). In early stages of osmotic stress due to the shortage of water in plant tissues, alfalfa plant reduces the growth of leaves and then decreases the development of shoot and reproductive growth (Farooq et al., 2015, 2017). Salt stress in alfalfa causes a decrease in rate of photosynthesis, as osmotic stress induces partial closure of stomata (Farooq et al., 2017). Absorption of sodium ions in the roots of alfalfa can be dangerous for its growth if present in cytosol at high concentrations (Assaha et al., 2017). High concentrations of sodium and chloride ions in the cytoplasm can disturb the cellular processes, also causes dehydration in cells as well as disturbs the process of photosynthesis (Munns and Tester, 2008; Ashraf and Harris, 2013). Increasing the ratio of cellular potassium to sodium as well as limiting the concentration of sodium in cytosol promotes salt tolerance in alfalfa cultivars (Sandhu et al., 2017). It is found that NaCl stress in alfalfa caused a significant increase in the activity levels of SOD, POD, CAT, and APX by 132.14%, 315.60%, 102.78%, and 27.61%, and a marked upregulation of two genes associated with salt stress (Chen et al., 2021).

Research has shown that stomatal opening can improve photosynthesis and biomass yield, but high concentrations of Na^+^ may cause stomatal closure, disrupting photosynthesis and causing an overproduction of reactive oxygen species (Kamran et al., 2020; Kimura et al., 2020; Adil et al., 2022a, 2023; Niu et al., 2022). In conditions of high salinity, Na^+^ in the apoplast surrounding the guard cells leading to stomatal closure (Kerstiens et al., 2002). A key mechanism to limit Na^+^ accumulation in the shoot is the reduction of transpiration rate by stomatal regulation (Yu and Assmann, 2016). The effect of Na_2_SO_4_ solution on alfalfa plants was studied from emergence to maturity, and reduction was recorded in relative emergence (%) at 12.7 dS m^-1^, with no survival of plants at 30 dSm^-1^ (Cornacchione and Suarez, 2015). The root growth of alfalfa is adversely less affected by salt stress as compared to that of shoot growth (Bertrand et al., 2020). Research was conducted on 15 populations of alfalfa under salt stress conditions, treated with a mixture of NaCl, Na_2_SO_4_, CaCl_2_ and MgSO_4_, and KCl, concluded that mass of root per plant at 18.4 dSm^-1^ and 24.5 ds m^-1^ electrical conductivity was decreased by 18% and 49% respectively whereas the recorded shoot mass reduction was nearly 50% and 73% (Cornacchione and Suarez, 2017). Alfalfa experienced a decline in biomass by 43%–86% and a 58%–91% decrease in nitrogen content; moreover, it negatively impacted nitrogen fixation and atmospheric nitrogen uptake by hindering nodule formation and decreased nitrogen fixation efficiency when salt levels surpassed 100 mmol Na_2_SO_4_ L^-^¹ (Wan et al., 2023). Salt (NaCl) applied at 9 dSm^-1^ reduced the size of leaf by 34%, mass of stem by 35% as well as height of plant by 32% respectively (Valizadeh et al., 2013).

### 4.2 Salt tolerance mechanisms

Salinity resistance in plant involves diverse mechanisms, such as production of osmolytes, stimulation of antioxidant defenses, acidification of the apoplast, ionic stability, and hormonal response regulation (Al-Farsi et al., 2020; Iqbal et al., 2023) (**Table 3**). Soil salinity interferes with ionic equilibrium in alfalfa, resulting in excessive buildup of Na^+^ and Cl^–^ in both roots and shoots (Rogers et al., 2003; Li et al., 2010). Thus, maintaining ionic balance under salt stress is crucial for enhancing salinity tolerance in alfalfa, which is crucial for regulating cell volume, sustaining membrane potential, and supporting enzymatic activities (Amin et al., 2021). Salt stress disrupts hormone levels, impacting osmotic regulation and photosynthesis, which ultimately hinders legume growth (Farooq et al., 2017). Primary plant hormones, namely auxins, gibberellins, ethylene, cytokinins, and abscisic acid (ABA), act as crucial regulators engaging various developmental signaling pathways in plants (Agudelo-Morales et al., 2021). Modifications in ABA and ethylene signaling have been observed in response to salinity stress (Farooq et al., 2017). and they are vital for salt tolerance (Sah et al., 2016; Nykiel et al., 2023). Higher ABA levels under salt stress stimulate stress protein production and induce osmotic regulation, hereby enhancing salt tolerance (Chen et al., 2022). The exogenous use of osmolytes and phytohormonal agents may reduce salinity-related losses in alfalfa (Deinlein et al., 2014). The accumulation of compatible solutes under stress conditions contributes to osmotic tolerance by both regulating intercellular osmotic pressure and protecting membranes from ROS damage (Deinlein et al., 2014; Farooq et al., 2015).

**Figure 4:**
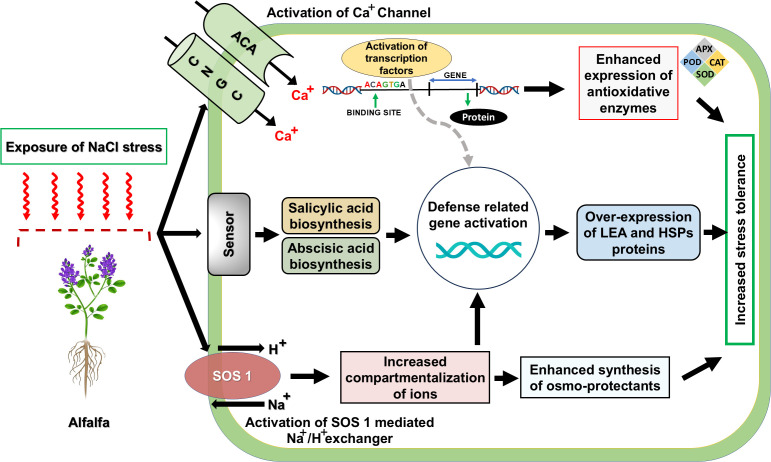
A Graphical Depiction of Plant Adaptations to Salinity Stress. CNGC: Cyclic nucleotide-gated channels, ACA, autoinhibited calcium ATPase; SOS, salt overly sensitive; Transcription factors (DREB; WRKY; NAC; MYB), Proteins (LEA, Late Embryogenesis Abundant proteins; HSPs, Heat Shock Proteins). Reproduced from Kashyap et al. (2021) Copyright 2021 Springer Nature.

The identification of salt-tolerant alfalfa lines has been extensively achieved by screening their resistance to salinity in various studies (Yu et al., 2021). Initiation of plant adaptation to saline conditions involves the early sensing of stress stimuli via molecular detectors like cyclic nucleotide-gated channels (CNGCs), autoinhibited calcium ATPases ACAs), as well as key regulators within the salt overly sensitive (SOS) network (Li et al., 2022), (**Figure 4**). Whereas the stress perception initiates signal transduction cascades, including salicylic acid and abscisic acid pathways, leading to the induction of multiple downstream genes and regulatory transcription factors (Bose and Howlader, 2020), (**Figure 4**). Latest findings demonstrated that several differentially expressed genes (DEGs) encode regulatory transcription factors such as DREB, NAC, WRKY, and MYB, that are believed to play a crucial role in transcriptional response to salinity stress in alfalfa (He et al., 2022), (**Figure 4**). Transcriptomic analysis revealed significant enrichment of heat shock proteins (HSPs), likely functioning within the MAPK signaling cascade, in salt-tolerant alfalfa, while marked upregulation of LEA family genes suggests their role in osmotic adjustment under salt (Hang et al., 2024), (**Figure 4**). Advances in molecular biology have made transgenic technology a popular and effective method for single trait improvement in plants, as compared to that of traditional breeding practices (Sun et al., 2024). For the development of salt-resistant alfalfa, the introduction of exogenous genes like the receptor kinase gene GsSRK (Sun et al., 2018), ZxNHX and ZxVP1-1 (Kang et al., 2016), thiamine thiazole synthase (THI1) gene MsTHI1 (Yin et al., 2022), Na^+^/H^+^ reverse transporter genes AtNHX1 (Stritzler et al., 2018), calcineurin B-like (CBL) gene MsCBL4 (An et al., 2020), and a rare cold-inducible 2/plasma membrane protein 3 (RCI2/PMP3) gene MsRCIs (Li et al., 2021) reported an improvement in salt tolerance in genetically modified alfalfa plants, aided by advancements in high-throughput sequencing and bioinformatics, coupled with transcriptomics, proteomics, and metabolomics has emphasized the vital role of transcription factors (TFs) (Ma et al., 2023; Zhang et al., 2023), metabolite biosynthesis and other abiotic genes related to stress resistance (Bhattarai et al., 2021; Kaundal et al., 2021), and miRNAs (Long et al., 2015; Ma et al., 2020b), are crucial for salt tolerance (Huang et al., 2020; Zhao et al., 2020). Compared to other crops, the mechanisms at the genetic, molecular, and physiological levels that confer salt resistance in alfalfa are still inadequately understood (He et al., 2022). Recent studies have shown that miR156 plays a key role in alfalfa’s response to salt stress by regulating the expression of target genes, including those coding for SPL protein family (Wang et al., 2021; Zhang et al., 2022a, 2022b, 2022c).

**Table 3:** Principal genes underlying alfalfa’s mechanism of salt stress tolerance.

Gene abbreviation	Full name of gene	Gene Functionality
*NHX1*	Na^+^/H^+^ exchanger 1	Regulation of Na^+^ accumulation in vacuole
*SGF29*	Transcriptional activator SaGa associated factor 29	Cellular signaling pathways
*GmDREB1*	Soybean DREB (dehydration-responsive-element binding protein) orthologue 1	Affects the osmolyte balance by regulating proline and sugar content
*P5CS1*	Delta1-pyrroline-5-carboxylate synthase 1	Improvement of antioxidants as well as accumulation of organic solutes
*GsZFP1*	Glycine soja putative Cys2–His2 type zinc finger protein	Reduces sodium influx and governs the synthesis and accumulation of proline
*HKT1*	High-affinity K^+^ transporter 1	Regulate mechanisms governing the retrieval of Na^+^ from xylem pathway
*rstB*	Putative sensor histidine kinase gene vda**_**000600	Restriction of Na^+^ uptake and regulation of calcium accumulation
*SOS1*	Salt Overly Sensitive 1	Prevention of Na^+^ ion entry into root tissues
*HSP81.2*	Heat-shock protein gene 81.2	Cellular signaling pathways
*AtNDPK2*	Arabidopsis nucleoside diphosphate kinase 2	Modulation of hydrogen peroxide-induced MAP (mitogen-activated protein) kinase signaling during osmotic stress responses
*OTSI*	Overlay tolerant to salt 1	Cellular signaling pathways
*BADH*	Betaine aldehyde dehydrogenase	Regulates osmolyte buildup and contributes to the maintenance of osmotic balance
*TPS-TPP*	Trehalose-6-phosphate synthase–trehalose-6-phosphate phosphatase fusion protein	Coordinates the metabolic pathways involved in trehalose accumulation
*WRKY20*	Probable WRKY transcription factor 20	Homeostasis of K^+^ and regulates accumulation of proline
*ERF1*	Ethylene response factor 1	Regulate ethylene and jasmonate signaling, to improve antioxidants and accumulation of organic solute
*MtNHX1*	*Medicago truncatula* Na^+^/H^+^ exchanger 1	Regulating ionic balance inside the vacuole

(Al-Farsi et al., 2020).

## 5 Conclusion and future perspectives

Environmental fluctuations and abiotic stress factors significantly disrupt agricultural productivity and reduce crop quality worldwide. As a vital forage legume valued for its substantial biomass production and rich nutritional profile, alfalfa remains susceptible to yield declines under abiotic challenges such as salinity, drought, and metal toxicity. Under these stress conditions, alfalfa engages complex regulatory systems at both the physiological and molecular levels, leading to changes in cellular structure, biochemical pathways, and transcriptional regulation. Despite advancements in model plant systems, significant gaps persist in our understanding of alfalfa’s molecular adaptations to stress, primarily due to its complex genome and outcrossing reproductive behavior, which make it a challenging experimental subject. In this review, we consolidate existing insights into the physiological adjustments and molecular adaptations of alfalfa under salinity, drought, and heavy metal stress conditions. These abiotic challenges activate intricate signaling cascades initiated at the cell wall or plasma membrane level through the perception of phytohormones, ions, and gaseous signaling molecules, leading to the regulation of subsequent stress-response pathways.

Deciphering the mechanisms by which alfalfa responds to stress is vital for enhancing breeding strategies focused on generating cultivars with improved tolerance to multiple environmental constraints. In this regard, numerous studies have identified diverse mechanisms of stress tolerance under controlled conditions, although findings from open-field experiments are still insufficient. Hence, to close this knowledge gap, implementing phenotyping at the cellular and tissue scale could offer new insights into how plants adapt to stress conditions, which can further boost our proficiency in designing stress-resistant cultivars of alfalfa. Furthermore, existing studies highlight those advancements in genetic transformation methods have led to the identification of several genes and signaling pathways related to stress resilience and adaptation. Nonetheless, considerable gaps persist regarding the identification of key genes, number of genes to target and understanding their specific involvement in regulating plant responses to stress. There is still an ongoing debate over whether all key genes are linked to particular stress conditions or if targeting a selective set of genes is a more effective strategy. This highlights the need for more comprehensive studies and cutting-edge approaches, such as single-cell omics, to pinpoint precise genetic elements crucial for enhancing stress tolerance in alfalfa.

No single strategy will suffice as plants often face multiple stresses simultaneously in natural environments. Thus, future studies should aim to examine the interactive impacts of multiple tolerance strategies and identify key genetic and biochemical pathways that can be targeted for breeding. Moving beyond conventional studies, we anticipate that a comprehensive approach combining genomics, bioinformatics, and functional genomics, focusing on the study of protein-nucleic acid interactions and gene regulation, is also crucial for exploring alfalfa’s genetic framework. These investigations will reveal new genes that can be linked to targeted traits, providing valuable insights to support the genetic enhancement of alfalfa. In addition, the application of modern molecular tools will play a vital role in advancing genetic manipulation methods including gene overexpression, precise gene editing, and *de novo* synthesis of genes for specific traits to strengthen stress adaptability and productivity.
